# Efficacy of prophylactic splenectomy for proximal advanced gastric cancer invading greater curvature

**DOI:** 10.1186/s12957-017-1173-9

**Published:** 2017-05-25

**Authors:** Yu Ohkura, Shusuke Haruta, Junichi Shindoh, Tsuyoshi Tanaka, Masaki Ueno, Harushi Udagawa

**Affiliations:** Departments of Gastroenterological Surgery, Toranomon Hospital, 2-2-2 Toranomon, Minato-ku, Tokyo, 105-8470 Japan

**Keywords:** Proximal gastric cancer, Prophylactic splenectomy, Greater curvature

## Abstract

**Background:**

For proximal gastric cancer invading the greater curvature, concomitant splenectomy is frequently performed to secure the clearance of lymph node metastases. However, prognostic impact of prophylactic splenectomy remains unclear. The aim of this study was to clarify the oncological significance of prophylactic splenectomy for advanced proximal gastric cancer invading the greater curvature.

**Methods:**

Retrospective review of 108 patients who underwent total or subtotal gastrectomy for advanced proximal gastric cancer involving the greater curvature was performed. Short-term and long-term outcomes were compared between the patients who underwent splenectomy (*n* = 63) and those who did not (*n* = 45).

**Results:**

Patients who underwent splenectomy showed higher amount of blood loss (538 vs. 450 mL, *p* = 0.016) and morbidity rate (30.2 vs. 13.3, *p* = 0.041) compared with those who did not undergo splenectomy. In particular, pancreas-related complications were frequently observed among patients who received splenectomy (17.4 vs. 0%, *p* = 0.003). However, no significant improvement of long-term outcomes were confirmed in the cases with splenectomy (5-year recurrence-free rate, 60.2 vs. 67.3%; *p* = 0.609 and 5-year overall survival rates, 63.7 vs. 73.6%; *p* = 0.769). On the other hand, splenectomy was correlated with marginally better survival in patients with Borrmann type 1 or 2 gastric cancer (*p* = 0.072).

**Conclusions:**

For advanced proximal gastric cancer involving the greater curvature, prophylactic splenectomy may have no significant prognostic impact despite the increased morbidity rate after surgery. Such surgical procedure should be avoided as long as lymph node involvement is not evident.

## Background

To date, concurrent splenectomy or distal pancreatectomy has been performed in selected cases with advanced gastric cancer to secure the clearance of potential nodal involvement at the splenic hilum or along the splenic artery. However, several recent retrospective studies have reported negative outcomes in prophylactic splenectomy and there have been discussions on the necessity of splenectomy for advanced gastric cancer [1–4].

For patients with advanced gastric cancer not involving the greater curvature of the stomach, a prospective study conducted by the Japan Clinical Oncology Group (JCOG0110) has confirmed that concurrent splenectomy has no oncological advantage despite the increased morbidity [5], and prophylactic splenectomy is currently not recommended for such cases in the Japanese Gastric Cancer Treatment Guidelines [6]. However, for advanced gastric cancer involving the greater curvature, concurrent splenectomy remains a standard procedure despite that no solid evidence has been available. Therefore, this study sought to clarify the prognostic impact of splenectomy for patients with proximal advanced gastric cancer involving the greater curvature of the stomach.

## Methods

### Study population

Initial cohort included 1309 patients who underwent total or subtotal gastrectomy for advanced proximal gastric carcinoma invading greater curvature at the Department of Gastroenterological Surgery, Toranomon Hospital, between January 1975 and December 2015. Among these, 108 patients were selected according to the following inclusion criteria: histopathologically proven adenocarcinoma, T2-4 in the upper 1/3 of the stomach invading the greater curvature, no gross nodal metastasis at the splenic hilum or along the splenic artery, no invasion to the spleen or the pancreas, and no evidence of distant metastases or peritoneal dissemination (both in macroscopic observation and peritoneal lavage). The definition of clinical lymph node-positive was enlarged lymph node (≧8 mm in the minor axis or ≧10 mm in the major axis) by contrast-enhanced abdominal computed tomography. The JCOG 0110 excluded Borrmann type 4 because of their aggressive malignant behavior. On the other hand, we included Borrmann type 4 because this type is thought to be associated with higher rate of peritoneal dissemination due to aggressive invasion of cancer cells to the gastric wall and it is a very important point that we should resect or preserve the spleen for these patients who had type 4 tumor. In the past report, a type 5 gastric cancer contained various types of tumors, from low-grade tumor to a high-grade tumor. However, the type 5 tumor generally has a large tumor diameter, high lymph node metastasis rate, and deeply invasive tumor. And also, these tumors has high rate of vascular invasion and high histological malignancy grade; therefore, Borrmann type 5 tumors were grouped with Borrmann types 3 and 4 [7, 8].

Then, the 108 patients were divided into two groups according to the concurrent splenectomy: the splenectomy group (*n* = 63) and spleen preservation group (*n* = 45), and clinical outcomes were compared.

### Surgery

D2 lymphadenectomy was routinely performed with total or subtotal gastrectomy for each patient. In the splenectomy group, lymphadenectomy along the splenic artery (No.11p and 11d lymph nodes) was completely performed with mobilizing the tail of the pancreas and only the spleen was excised while preserving the pancreatic parenchyma. In the spleen preservation group, No.11p and 11d lymph nodes were dissected without mobilizing the pancreatic tail and spleen. Lymph nodes at the splenic hilum (No.10 lymph nodes) were left untouched, but were dissected if judged easily removable in lean patients. In our hospital, treatment of advanced proximal gastric cancer was total gastrectomy with simultaneous splenectomy for adequate regional lymphadenectomy, especially splenic hilar lymph nodes. However, these indications were not established and so the surgeons finally determined whether it should be performed with or without splenectomy.

Clinical stage was determined according to the Union for International Cancer Control TNM Classification of Malignant Tumors, 7th edition on pathological examination [9]. A diagnosis of postoperative complications was made when we observed adverse events that corresponded to grade 2 or greater in the Clavien-Dindo classification [10].

### Data analyses

Pairwise differences of proportions and medians were analyzed by the chi-squared test, Fisher’s exact test, or Mann-Whitney *U* test, as appropiate. Cumulative overall survival rates (OS) and recurrence-free survival rates (RFS) were analyzed by the Kaplan-Meier method. All statistical analyses were performed using Statistical Package for the Social Sciences (SPSS) version 19.0J for Windows (SPSS Inc., Chicago, IL). This study was approved by the Internal Review Board of Toranomon Hospital.

## Results

### Patient characteristics

Table 1 shows the clinicopathological characteristics of the patients. No significant intergroup difference was observed in clinical findings such as age, sex, tumor depth, and macroscopic or histological type. Operative duration and curability of surgery did not differ between the groups, whereas the amount of blood loss was significantly higher in the splenectomy group. As for the pathological findings, lymphatic and venous invasion, nodal metastasis, and pathological stage and postoperative chemotherapy did not differ significantly between the groups. In the spleen preservation group, No.10 dissection or sampling without splenectomy was performed in 11 patients (24%) and the median number of retrieved No.10 nodes in these patients was 3. There were no lymph node metastases. In the splenectomy group, four patients (6%) were histopathologically diagnosed with nodal involvement at the splenic hilum. Of these four patients, two patients developed recurrence in peritoneum and two patients have no recurrence. The mean survival time of these four patients was 62.0 months.

**Table 1:** Clinicopathological characteristics of the 102 patients

	Splenectomy (*n* = 63)	Spleen preservation (*n* = 45)	*p* value
Clinical findings			
Age, median (range)	64.0 (40–87)	65.6 (20–89)	0.416
Sex			0.135
Male	39	34	
Female	24	11	
Depth of invasion			0.769
cT2	29	22	
cT3–4	34	23	
Clinical N factor			0.625
cN(−)	31	20	
cN(+)	32	25	
Borrmann macroscopic type			0.196
Type 1 or 2	18	8	
Type 3, 4, or 5	45	37	
Histopathological type			0.406
Intestinal	23	20	
Diffuse	40	25	
Operative findings			
Operative duration (min)	297	277	0.660
	(165–440)	(140–504)	
Blood loss (ml)	538	450	*0.016*
	(152–1900)	(10–1052)	
Curability			0.973
Cur A	24	17	
Cur B	39	28	
Pathological findings			
Lymphatic invasion			0.512
Negative	16	9	
Positive	47	36	
Venous invasion			0.267
Negative	10	11	
Positive	53	34	
Lymph node metastasis			0.746
pN0	23	16	
pN1	13	12	
pN2,3	27	17	
Pathological stage			0.165
p stage I	9	2	
p stage II	18	21	
p stage III	36	22	
Postoperative chemotherapy			0.388
Yes	49	38	
No	14	7	

### Postoperative complications

Table 2a compares the postoperative complication between the two groups. Splenectomy group showed higher rate of grade 2 or greater complications according to the Clavien-Dindo classification system (30.2 vs. 13.3%, *p* = 0.041). The rate of pancreas-related complications was particularly higher in the splenectomy group (17.4 vs. 0%, *p* = 0.003), while the rates of other types of complications were almost equivalent between the groups.

**Table 2:** Postoperative complications and recurrence rates/ patterns

	Splenectomy (*n* = 63)	Spleen preservation (*n* = 45)	*p* value
(a) Postoperative complications			
Morbidity (CD grade 2 or higher)	19 (30.2%)	6 (13.3%)	*0.041*
Pancreatic related	11	0	*0.003*
(Pancreatic fistula/abdominal abscess)	(17.4%)	(0%)	
Ileus	2	1	0.767
Anastomotic leakage	2	3	0.395
Postoperative bleeding	1	1	0.809
Anastomotic stenosis	2	0	0.261
Other	1	1	0.809
(b) Postoperative outcome			
Number of recurrence	22 (34.9%)	15 (33.3%)	0.864
Borrmann macroscopic type			0.538
Type 1 or 2	4	4	
Type 3, 4, or 5	18	11	
Main recurrent patterns			
Peritoneal dissemination	17 (27.0%)	9 (20.0%)	0.403
Distant metastasis (Liver, lung, abdominal wall)	3 (4.8%)	4 (8.9%)	0.390
Lymph node metastasis	2 (3.2%)	2 (4.4%)	0.731

*CD* Clavien-Dindo classification

### Outcomes after gastrectomy

**Fig. 1:**
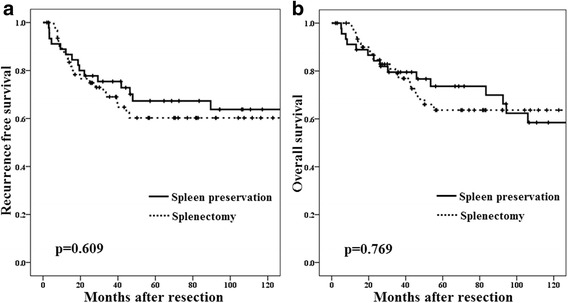
**a** Recurrence-free survival curve. Five-year recurrence-free (RFS) rates did not differ significantly between the splenectomy and spleen preservation groups (60.2 vs. 67.3%, *p* = 0.609). **b** Overall survival curve. The 5-year OS was 73.6% in the spleen preservation group and 63.7% in the splenectomy group (*p* = 0.769)

**Fig. 2:**
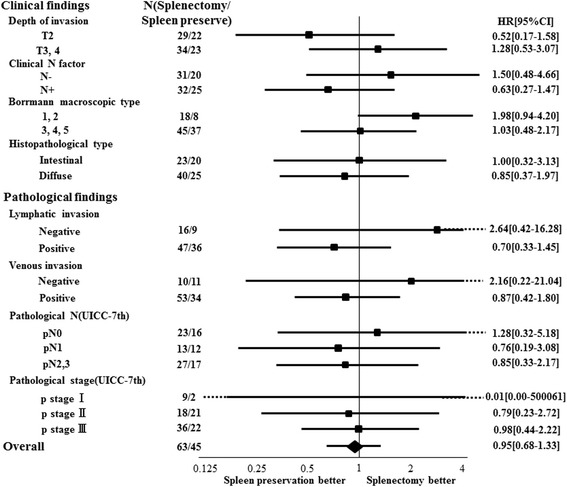
Subset analysis. Splenectomy was correlated with marginally better survival in patients with Borrmann type 1 or 2 gastric cancer (*p* = 0.072)

The median follow-up period was 135 months in the splenectomy group and 189 months in the spleen preservation group (*p* = 0.188) (Kaplan-Meier estimate). Table 2b shows recurrence rates and patterns, and RFS curves are shown in Fig. 1a. Five-year recurrence-free (RFS) rates did not differ significantly between the splenectomy and spleen preservation groups (60.2 vs. 67.3%, *p* = 0.609). As for the recurrence patterns, peritoneal dissemination occurred in 28.6% of patients in the splenectomy group and 22.2% in the spleen preservation group, with no significant intergroup difference. Isolated recurrence of nodal metastasis at the splenic hilum was observed in two (4.4%) patients in the spleen preservation group. Overall survival (OS) curves are shown in Fig. 1b. The 5-year OS was 73.6% in the spleen preservation group and 63.7% in the splenectomy group (*p* = 0.769). In a subset analysis, splenectomy was correlated with marginally better survival in patients with Borrmann type 1 or 2 gastric cancer (*p* = 0.072). No other subgroups showed potential advantage of splenectomy for OS (Fig. 2). In addition, of all 37 patients with recurrence, the rate of the peritoneal dissemination in patients with Borrmann type 1 or 2 (4 out of 8 patients; 50.0%) was lower than in patients with Borrmann type 3, 4, or 5 (24 out of 29 patients; 82.6%), but the difference between the two groups was not significant (*p* = 0.056).

### Outcomes after therapeutic splenectomy

For reference, 26 patients who were not included in this study due to therapeutic splenectomy for evident nodal involvement in No.10 or No.11p and 11d lymph nodes were additionally analyzed. Table 3 shows the clinicopathological characteristics of these patients stratified by tumor recurrence. Of the 26 patients, 17 (65%) patients developed recurrence in the peritoneum (*n* = 16) or para-aortic node (*n* = 1). The median survival time of these 17 patients was 22.9 months, while the remaining 9 patients were alive without recurrence with a median survival time of 140.9 months. No evidence difference was observed in clinicopathological findings between the patients who developed recurrence and those who did not.

**Table 3:** Outcome after therapeutic splenectomy

	Recurrence (*n* = 17)	No recurrence (*n* = 9)	*p* value
Clinical findings			
Age, median (range)	59.0 (38–78)	60.0 (43–66)	0.358
Sex			0.484
Male	7	5	
Female	10	4	
Borrmann macroscopic type			0.463
Type 2	1	2	
Type 3	7	3	
Type 4 or 5	9	4	
Histopathological type			0.778
Intestinal	3	2	
Diffuse	14	7	
Operative findings			
Operative procedure			0.143
Splenectomy	3	4	
Splenectomy + pancreatectomy	14	5	
Curability			0.161
CurA	0	1	
CurB	17	8	
Final stage			0.359
Stage IIIA	2	2	
Stage IIIB	12	7	
Stage IIIC	3	0	
Lymph node metastasis at the splenic hilum	17	9	
Lymph node metastasis along the splenic artery	5	4	
Median survival period (months)	22.9	140.9	<0.001
Main recurrent patterns			
Peritoneal dissemination	16 (94.1%)		
Lymph node metastasis	1 (5.9%)		

## Discussion

Concurrent splenectomy is rarely performed in Western countries because it is reportedly associated with increased risk of postoperative complications with no survival benefits [11, 12]. The JCOG 0110 study, a prospective randomized comparative study conducted in Japan, reported that prophylactic splenectomy has no prognostic advantage in patients with proximal advanced gastric cancer not invading the greater curvature of the stomach [5]. However, it remains inconclusive whether splenectomy is needed or not in patients with proximal advanced gastric cancer involving the greater curvature of the stomach, which would be a risk factor for nodal involvement around the splenic hilum. The current study focused on the specific population with proximal gastric cancer involving the greater curvature of the stomach and revealed that splenectomy was associated with increased risk of morbidity, and no prognostic advantage except for the specific subpopulation with Bormann type 1 or 2 tumor.

From the standpoint of safety of surgery, concurrent splenectomy was associated with significant increase in blood loss and postoperative complications in this study. The overall rate of postoperative morbidity was similar with those in the prospective JCOG0110, and the current study clarified that splenectomy was associated with significantly higher rate of pancreas-related complications (*p* = 0.003) such as pancreatic fistula/abdominal abscess [13, 14]. Mobilization of the pancreas from the retroperitoneum and extensive lymphadenectomy along the splenic vessels may be associated with these clinical results in the splenectomy group [15, 16]. Given that the rate of nodal involvement in the dissected lymph nodes was only 6%, oncological relevance of the extensive lymphadenectomy is questionable and the current results may support the avoidance of prophylactic splenectomy even in the cases with proximal advanced gastric cancer located at the greater curvature of the stomach.

In previous retrospective studies, regardless of tumor location, the rates of 5-year survival were ≥10% lower in the splenectomy group than those in the spleen preservation group [4, 17–19]. In the present study, however, no significant prognostic difference was confirmed between the splenectomy group and the spleen-preserving group in both 5-year RFS (60.2 vs. 67.3%) and 5-year survival rates (63.7 vs. 73.6%). Recurrence after spleen-preserving surgery in the corresponding lymph nodes which will be excised when splenectomy is performed was observed only in two (4.4%) patients. Furthermore, the patterns of recurrence were similar regardless of the splenectomy, with relatively high proportion of peritoneal dissemination (70–80%) among all recurrent sites in the study group. These results may also support that prophylactic splenectomy is unnecessary for most of the cases with proximal advanced gastric cancer.

Of note, however, subset analysis stratified by the macroscopic subtype of gastric cancer showed different tendencies of prognostic advantage in splenectomy between Borrmann type 1 or 2 and Bormann type 3, 4, or 5. The latter subtypes of gastric cancer is thought to be associated with higher rate of peritoneal dissemination due to aggressive invasion of cancer cells to the gastric wall, and as expected, there was no prognostic advantage of splenectomy due to high rate of peritoneal recurrence [20]. Meanwhile, Borrmann type 1 or 2 is mass-forming subtypes with less aggressive invasion of cancer cells into the stomach wall. The subanalysis showed that the splenectomy group for Borrmann type 1 or 2 may be associated with marginal prognostic advantage (Hazard ratio, 1.98; 95% CI, 0.94–4.20; *p* = 0.072). Actually, the two patients who had initial recurrence in the lymph node at the splenic hilum had Bormann type 1 or 2 tumor with no evidence of peritoneal dissemination.

From the practical standpoint, the current results suggest that prognostic advantage of splenectomy is relatively small in the overall population with proximal advanced gastric cancer due to aggressive patterns of recurrence including peritoneal dissemination. However, the subanalysis suggested a potentially encouraging result in a specific subpopulation with Borrmann type 1 or 2 tumors. Given that 35% of the patients with definitive findings of mesenteric infiltration or nodal metastasis at the splenic hilum or along the splenic artery (the reference cohort in this study) achieved median recurrence-free survival of 140.9 months after therapeutic lymphadenectomy; splenectomy with extensive lymphadenectomy may be effective for selected population with locally advanced cancer involving the lymph nodes. Therefore, prophylactic splenectomy with extensive lymphadenectomy remains a potentially favorable surgical approach for patients with Borrmann type 1 or 2.

The limitation of this study includes its retrospective nature and selected population. However, perioperative management was similar during the study period, and the current study is based on a prospectively collected database for consecutive patients. Although prophylactic splenectomy showed no significant prognostic advantage in the overall population, the subpopulation with Borrmann type 1 or 2 might remain a candidate for splenectomy with marginal improvement of overall survival after splenectomy with relatively low rate of peritoneal recurrence. An external validation study using a sufficient number of patients would be needed to confirm the current observations.

## Conclusions

Prophylactic splenectomy in advanced proximal gastric cancer invading the greater curvature increases postoperative complications without clearly improving RFS or OS, indicating that the procedure is not effective from a viewpoint of risk-benefit balance. However, in a subgroup of patients with less invasive macroscopic subtypes of gastric cancer, splenectomy might have a potential advantage for local control of tumor and further investigation is needed.

## Abbreviations

OS: Overall survival rates
RFS: Recurrence-free survival rates
